# Implementation of an interprofessional medication adherence program for chronic patients in community pharmacies: how much does it cost for the provider?

**DOI:** 10.1186/s12913-018-3851-x

**Published:** 2019-01-08

**Authors:** Clemence Perraudin, Jean-François Locca, Christophe Rossier, Olivier Bugnon, Marie-Paule Schneider

**Affiliations:** 1 Department of Ambulatory Care and Community Medicine, Community Pharmacy Center, University of Lausanne, Lausanne, Switzerland; 2 Pharmacie de Prilly, Community Pharmacy, Prilly, Switzerland; 3 Pharmacie de l’Ile, Community Pharmacy, Rolle, Switzerland; 4 Community Pharmacy Practice Research, School of Pharmaceutical Sciences, University of Geneva, Geneva, Switzerland

**Keywords:** Implementation, Medication adherence, Community pharmacy, Cost analysis, Break-even analysis, IMAP

## Abstract

**Background:**

The implementation of an innovative and sustainable professional pharmacy service in routine care requires substantial resources borne by the pharmacy owner. Although a community pharmacy is a business setting, few studies have examined cost as a potential barrier to widespread implementation. Implementation costs, as the cost impact of an implementation effort, can be significant and hamper the decision to invest from the provider perspective. Traditional financial planning tools can be used to analyse and support business decision to implement a service by assessing the net impact of a new service on the provider’s budget. This study aimed to estimate the implementation costs and the break-even point of an interprofessional medication adherence program for chronic patients in Switzerland. The program combines motivational interviews, medication adherence electronic monitoring and feedback reports to patient and physicians.

**Methods:**

We used a 3-step approach: (i) micro-costing analysis: identification of implementation activities, quantification and valuation of required resources. Implementation costs, including service support costs and direct delivery costs, were analysed according to the implementation phase (installation, initial implementation, and full operation); (ii) break-even analysis: estimation of the required number of patients to follow up with to ensure that the generated revenue exceeded the total cost; and (iii) univariate sensitivity analyses.

**Results:**

The estimated total cost of the installation phase was 8481 CHF, more than half of which represented the cost of the equipment. Direct delivery costs were 666 CHF per patient per year, with 68% of this value associated with the cost of workforce time. According to the Swiss national reimbursement system, a minimal of 16 [10–27] patients was required to cover the implementation costs of the installation phase. This break-even point decreased to 13 patients in the initial and full operation phases.

**Conclusions:**

These estimates lead to a better understanding of the real cost of implementing a professional pharmacy service in routine care. In a Swiss context, the current medication adherence support fee-for-service system allows pharmacists to reach the break-even point. Such information is important for community pharmacists to guide their implementation strategies. The replication of similar analyses in other settings and countries is paramount.

## Background

In 2011, the World Health Organization (WHO) and the International Pharmaceutical Federation (FIP) officially adopted changes in the practices of community pharmacists from the delivery of medicines to a collaborative, person-centred care process [1, 2]. In practice, a variety of professional pharmacy services are now available in pharmacies, ranging from screening services to prescription renewals, medication reviews, and adherence-enhancing services. From a healthcare system perspective, some of these services are effective [3–7] and cost-effective in experimental contexts [8] and can increasingly be charged as fees-for-services [9, 10].

Medication nonadherence is endemic and decreases the cost-effectiveness of chronic treatments [11–13]. In 2004, the Community Pharmacy Center of the Department of Ambulatory Care and Community Medicine (Policlinique Médicale Universitaire, PMU), University of Lausanne (Switzerland) developed and implemented an interprofessional medication adherence program (IMAP) for chronic patients. It consists of a multifactorial, semi-structured intervention combining individualised, short but repeated motivational interviews, medication adherence electronic monitors (EMs) (MEMS, Aardex MWV, Switzerland) and feedback to the patient. A secure web platform allows the pharmacist to record the patients’ data, provide adherence feedback to the patient during the interview, report to the physician and other professionals, and use the electronic records to guide the intervention from one interview to the next one. The program has been described in detail elsewhere [14, 15]. For example, in 2014, 268 patients were followed up by the Community Pharmacy Center: 187 HIV patients, 28 multiple sclerosis patients, 9 oncology patients and 44 patients with various chronic diseases (e.g., hypertension, type 2 diabetes and chronic dialysis) [15]. Currently, the program is being implemented in some voluntary community pharmacies in the French-speaking part of Switzerland for HIV patients [16], multiple sclerosis patients [17] and patients with type 2 diabetes [18]. Published literature on the program in HIV patients showed an increased medication adherence and persistence among participants compared to a control group at 6 months (quasi-experimental study, *n* = 32) [19]. Moreover, the number of undetectable HIV patients increased during the 11-month program (retrospective analysis, *n* = 104) [14]. Finally, the program significantly improved retention in care at 6 and 12 months (retrospective analysis, *n* = 762) [20]. Other findings in the literature assessing the effectiveness and cost-effectiveness of interventions to improve adherence in HIV and chronic patients corroborate these positive results [21–23].

Despite these encouraging developments, the implementation of professional pharmacy services in routine care faces certain challenges [24–26]. Among other factors investigated in the literature, cost is a potential barrier to widespread implementation [27–29]. Implementation costs is defined as the cost impact of an implementation effort [28]. They can be significant and may hamper the provider’s decision to invest [30, 31]. They occur during three different phases of the implementation process [32–34]: installation phase (i.e., the preparation of the pharmacy and service provider to deliver the service), initial implementation phase (aimed to experiment the service provision to a small number of patients) and full operation phase (i.e., the full implementation and the provision of the service in routine care) [32]. Within these phases, two components of implementation costs can be distinguished [31]: direct service delivery costs and service support costs. First, direct service delivery costs vary with each additional patient (i.e., variable costs) and include all costs associated with the delivery of the service, primarily labour costs. Second, service support costs occur both in the installation phase, as start-up costs (e.g., initial staff training, equipment), and in the implementation and full operation phases (e.g., continuous training, supervision meetings). These costs are independent of the number of patients (i.e., fixed costs). Finally, the true implementation cost of a professional pharmacy service notably depends upon the complexity of the service, the complexity of the used implementation strategies, and the setting of service delivery (complexity and overheads) [28].

The literature often focuses on the direct service delivery costs, and rarely includes service support costs, or it values at best the initial staff training. However, the implementation of a sustainable, innovative service requires substantial resources to organise, engage and integrate the pharmacy in a novel philosophy of practices and business strategy. To be financially feasible and viable, the total revenue generated by the implementation of the service must at least cover the total cost of its implementation. Hence, traditional financial planning tools can help to analyse and support the business decision to implement a service by assessing the net impact of a new service on the provider’s budget [35]. The break-even analysis (BEA) can identify the price of a service and/or determine the volume of service needed to break even financially [36]. In a context of a fee-for-services fixed price, BEA is interesting to identify the required number of patients to follow up with (“the break-even point”) to ensure that the generated revenue will exceed the total cost.

This pragmatic study aimed to estimate implementation costs and the break-even point (in terms of the number of patients to follow up with) based on a real-world activity of the interprofessional medication adherence program for chronic patients.

## Methods

### Cost analysis

This analysis assessed the costs of a community pharmacy to implement the program. Because it focused on the implementation costs from the provider’s perspective, patient and societal costs were not considered. We performed a micro-costing analysis that included three steps [37, 38]: (i) identifying the relevant activities necessary to implement, maintain and deliver the program, (ii) quantifying the required resources for each activity (e.g., labour, space, material), and (iii) valuing a unit cost for each resource. The analysis is presented in accordance with the project management tool: PERT chart (Program Evaluation and Review Technique) used to schedule, organize, and coordinate tasks within the project. The cost estimates were expressed in Swiss Francs (1 CHF = 0.88€ = $0.99, http://www.xe.com, 11/13/2018). The initial staff training and investment, as well as the equipment, were amortisable over a 5-year amortisation period.

### Identification of activities & quantification of required resources

**Fig. 1:**
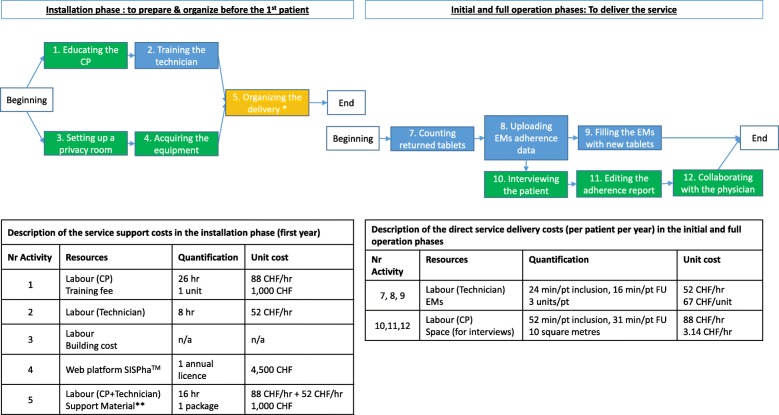
Activities and associated cost items to implement and deliver the program (PERT graph).  Activities performed by the community pharmacist.  Activities performed by the pharmacy technician.  Activities performed by both of them. CHF: Swiss Francs; CP: community pharmacist; EMs: electronic monitors; FU: follow-up: hr.: hour; min: minutes; n/a: not applicable; pt.: patient. * Including: writing the organizational procedures, preparing the support material, participating to coaching meetings, lauching the local networks and training in the web platform. ** Including patient leaflets, information and education material

The identification of the relevant activities and the quantification of required resources were based on the experience of the Community Pharmacy of the PMU, University of Lausanne (Switzerland) [15]. The cost estimates were validated by consensus with two external community pharmacy owners established in the same Swiss canton (canton de Vaud) (JFL, CRO). Implementation costs occurring in the different phases of the implementation process (installation, initial and full operation phases) were differentiated by service support costs and direct service delivery costs according to the classification of Garcia-Cardenas et al. (2016) (see Fig. 1). Service support costs in the installation phase corresponded to start-up costs, required before the inclusion of the first patient.

### Service support costs

#### Professional training: Pharmacist’s education

During the installation phase, the initial pharmacist training focused on (1) knowledge of medication adherence and (2) motivational interviewing skills. First, the pharmacist attended a 10-h adherence course organised into 5 modules: (i) introduction to medication adherence, (ii) theoretical frameworks of medication adherence, (iii) medication adherence interventions, (iv) structure of medication adherence interviews, and (v) case studies. Second, the pharmacist was trained in motivational interviewing during four 4-h sessions [15]. Then, during the initial and full operation phases, the pharmacist attended one day of continuous education per year, consisting of an update on medication adherence or motivational interviewing. For instance, at the Community Pharmacy Center of the PMU, a 1-h adherence internal meeting is organised every 6 to 8 weeks for educational purposes and for discussing complex case studies [15]. In the analysis, we considered the labour cost (i.e., time spent on training) and the course registration fee.

#### Professional training: Pharmacy technician’s training

In the analysis, we considered a day of internal training per each new pharmacy technician during the installation phase. The objectives were to train technicians on the handling of EMs, the secure uploading of EMs data and counting tablets. Moreover, during the initial and full operation phases, the technicians participated in the same education internal meetings along with the pharmacists. Initial training costs were amortised over 5 years.

#### Implementation strategies

In the installation phase, the pharmacist-technician duo needed two days to prepare and organise the delivery of the service. This time included the development of the planning process, the writing of procedures and the preparation of support material. To ensure the quality of the service and the fidelity in its delivery, the service is ISO certified at the Community Pharmacy Center of the PMU and all procedures are written and made available to the team at any time. The cost of support material was estimated by expert opinion, including patient leaflets and information and education material. In the initial and full operation phases, these efforts decreased but were still considered.

#### Equipment

According to Swiss law, a community pharmacy who offers a professional pharmacy service must have a privacy room or area that allows confidentiality for a face-to-face interview with the patient. Based on this fact, we assumed that pharmacists already had this space (including a computer) available for the medication adherence program, hence their acquisition costs were not considered in the analysis. As part of the equipment, we included the cost of the licence for a secure web platform to collect electronic patient data (i.e., SISPha®). This licence also comprises the implementation and technical support, including coaching meetings to guide the pharmacist during the interview and to edit the report for the physician and the nursing team (http://www.sispha.com). The cost of this annual licence was considered during all three phases of the implementation process.

### Direct service delivery costs

The average duration of the follow-up per patient was estimated at one year in accordance with PMU data (median: 333 days; IQR_25_: 138; IQR_75_: 799) [15]. The frequency of interviews with the pharmacist depends on the patient’s needs, with a more intensive support activity at the beginning of follow-up. Hence, this analysis considered the theoretical frequency of interviews starting at once a month for the first three months and then once a trimester thereafter (associated with prescription refill). Accordingly, we estimated one inclusion and six follow-up visits per patient for the first year.

#### Workforce time

The variable costs associated with the delivery of the service included the pharmacist time (patient interviews, adherence report editing, collaboration with the physician) and the technician time (EMs handling). Since 2011, the Community Pharmacy Center of the PMU routinely collects all time, except for coordination. Thus, we used these established median durations [15]. However, because these data are self-reported and from a program in operation since 2004, they are affected by the professional’s experience and may also be underestimated. Therefore, in accordance with experts, we added 20% to the original PMU time data to more precisely estimate the real time needed for implementing the service in inexperienced community pharmacies. Additionally, we evaluated the time required for the coordination, including time spent on launching local networks between the pharmacist and the physicians and all interprofessional contacts during a patient follow-up (estimated by expert opinion).

#### Electronic monitoring

Health insurance in Switzerland effectively reimburses the medication adherence program for all polypharmacy patients, defined as people who are simultaneously prescribed at least three chronic treatments and who are referred by their physician to their pharmacist because of a history or a risk of poor adherence. Therefore, in this analysis, we decided to include the costs of three EMs per patient. The EM unit cost was annualised to consider a battery life of two years, including the cost of the hardware required to read EMs data.

### Valuation of unit cost for each resource

#### Labour cost

The hourly labour costs for health professionals were estimated by expert opinion based on the Swiss salary scale in the canton of Vaud (http://www.vd.ch). This scale defines a salary level per profession (classes ranging from 1 to 18) according to professional experience (grades ranging from 0 to 26). The annual gross salary for a mid-career professional corresponded to class 11/grade 13 for a community pharmacist and class 4/grade 13 for a pharmacy technician. According to the finance department at the PMU, a multiplier coefficient (22%) was applied to consider social security contributions and reflect the full cost to the employer. Finally, the number of annual working hours used corresponded to the usual number of effective working hours in Switzerland (42 h30 per week excluding holidays, absenteeism and downtime).

#### Space cost

We considered the opportunity cost (i.e., the value of the resource in an alternative use) of the interview private room/confidentiality area used for the delivery of the service. To estimate the hourly cost of this space, the size of the room was fixed by expert opinion to ten square metres. The cost per square metre included the annual rent, maintenance and utility fees, derived from the annual cost survey on Swiss pharmacies in 2014 (“RoKA” report, http://www.pharmasuisse.org/de/). The total annual cost was divided by the mean opening hours in the canton (57 h per week in the “RoKA” report).

### Break-even analysis (BEA)

**Fig. 2:**
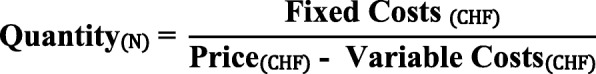
Break-even analysis (BEA) formula

The BEA formula can determine the volume of services (i.e., quantity, expressed in terms of the number of followed-up patients) needed to ensure that the generated revenues exceed the costs [36] (see Fig. 2). The “price” corresponded to the fixed total revenue per patient (i.e., the total fees charged by the pharmacist to the Swiss health insurances, depending on the number of chronic medications taken by the patient). In our case, it included a medication adherence support fee-for-service (21.60 CHF per week) and the sale of a pill organiser (18 CHF per quarter) per patient (http://www.pharmasuisse.org/de/). We estimated the break-even point using Microsoft Office Excel 2007 software.

### Sensitivity analyses

Univariate sensitivity analyses were performed to assess the impact of the uncertainty generated by estimated parameters (other things being equal). This uncertainty takes into account the risk management of the activity (e.g. longer interview time with complex patients). Only the parameters estimated to have the greatest impact on the model output were assessed:

(i) the professional delivery time using interquartile ranges (IQR_25_, IQR_75_) [15];

(ii) the pharmacist and technician labour costs using +/− 20% of the base case values to account for the impact of professional experience level;

(iii) the number of EMs per patient using one and four EMs for the minimum and the maximum scenarios; and (iv) the doubling of trained professionals to ensure a full-time capacity to deliver the program.

## Results

In the installation phase of the implementation process, the total cost was estimated at 8481 CHF, more than half of which represented the cost of the equipment (see Table 1). Service support costs were lower in the initial and full operation phases. The variable costs (i.e., direct service delivery costs) were 666 CHF per patient per year, with 68% representing the cost of workforce time.

**Table 1:** Estimation of implementation costs

	Cost (CHF)	Contribution (%)
Installation phase		
(1) Service support costs	8481	100%
Initial training*	741	9%
Implementation strategies	3240	38%
Organisation	2240	26%
Support material **	1000	12%
Equipment	4500	53%
Initial and full operation phases		
(1) Service support costs (per year)	6680	100%
Continuous training	1120	17%
Implementation strategies	1060	16%
Organisation	560	8%
Support material **	500	7%
Equipment	4500	67%
(2) Direct service delivery costs (per patient per year)	666	100%
Workforce time	455	68%
Pharmacist	353	53%
Technician	102	15%
Electronic monitoring (EMs)	201	30%
Space	10	2%

*Amortised over 5 years

^**^Including patient leaflets, information and education material

CHF = Swiss Francs

**Fig. 3:**
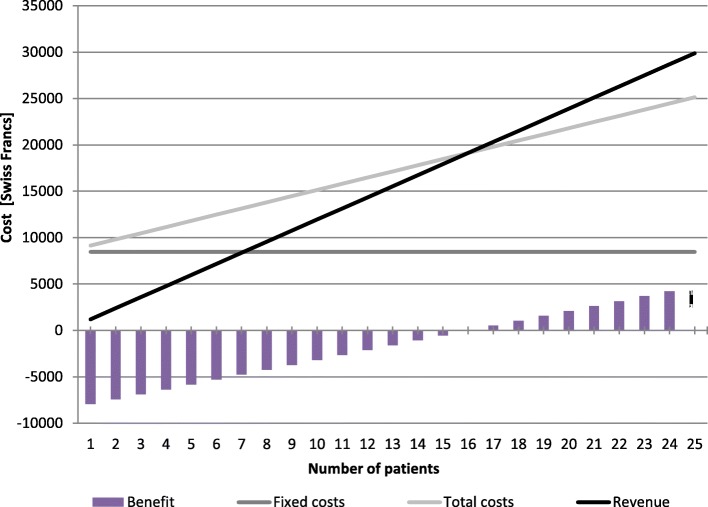
Break-even analysis

According to the national reimbursement system and the cost estimation, a minimal of 16 patients was required to cover the implementation costs of the installation phase (see Fig. 3). In this case, service support costs represented 39% of total costs. The break-even point decreased to 13 patients in the initial and full operation phases. The sensitivity analyses estimated the break-even points to be between 10 and 27 patients, according to the implementation phase and the scenario (see Table 2).

**Table 2:** Sensitivity Analyses

	Base case	Professional cost		Professional delivery time		Number of EMs		Doubling of trained professionals
	–	Min	Max	Min	Max	Min	Max	
Installation phase								
(1) Service support costs	8481	7971	9106	8481	8481	8481	8481	9222
Initial and full operation phases								
(1) Service support costs	6680	6344	7016	6680	6680	6680	6680	7800
(2) Direct service delivery costs	666	575	757	508	881	532	733	666
Revenue	1195.2	1195.2	1195.2	1195.2	1195.2	1195.2	1195.2	1195.2
BEA (year 1)	16	13	21	12	27	13	18	17
BEA (year 2 onwards)	13	10	16	10	21	10	14	15

EMs: medication adherence electronic monitoring

## Discussion

These estimates provide a better understanding of the real cost of implementing an interprofessional medication adherence program for chronic patients in Swiss community pharmacies. Due to the heterogeneity in interventions, data collections, item costs, or national professional costs, it is difficult to directly compare with other similar studies [32, 39]. However, all these studies consistently indicated that service support costs in the installation phase are substantial and that professional costs are the primary drivers of direct service delivery costs.

Our study is the one of the first to estimate the service support costs, distinguished during the implementation phases. Their significance supports the development of their valuation in cost analysis and economic evaluation. Service support costs, as described in this analysis, are required to guarantee the delivery of a high and standardised quality level service, but could also hamper the decision to invest from the provider perspective. In Switzerland, pharmacists’ training in advanced patient-oriented services is part of their postgraduate or continuous education. Today, the costs of such trainings are therefore borne by the pharmacy owners. However, this could change over time in accordance with the current curricula reform in Switzerland. One of the objectives of this reform is to strengthen in the pre-graduated cycle the clinical skills of pharmacists to deliver advanced patient-oriented services in the pre-graduated cycle. The curricula content influences the dissemination of professional pharmacy services and must accompany the evolution of the profession, according to national needs. Moreover, the specific education required to deliver the program can generate indirect revenues in other fields of business, allowing the pharmacist to invest time and skills in communicating with patients at risk for nonadherence.

Variable costs were primarily driven by workforce time (68%). We assume that this time will decrease and stabilise in the medium term with routine program delivery [32], providing a better profitability. Space cost was not very high. However, this valuation considers the opportunity cost associated with the use of a private area for the delivery of the program. Assuming that this area already existed, the growing number of patients followed could disrupt its previous use. Business diversification, including new professional services, affects the spatial organisation at the community pharmacy, notably associated with the offer of activities with and without appointments.

With our assumptions and the current national reimbursement system model, a minimum of 16 patients must be follow up with to ensure a profit for the pharmacy, with a range from 10 to 27 according to scenarios assessed in sensitivity analyses. In real life, some patients may stop the follow-up before one year, and the frequency of interviews will depend on the patient’s needs. Is this number realistic in terms of inclusion and follow-up rates? The patient inclusion process into the medication adherence program depends on several factors, which are specifically linked to the setting: the needs of the targeted population of patients, the state of the interprofessional collaboration, and the pharmacists’ ability to include patients. First, about 50% of chronic patients do not take their medications as prescribed [40]. Each professional has a role to play to support such patients in the management of their medications, notably the community pharmacist, as the first port of call in primary health care and the specialist of medicines. Due to the endemic nature of medication nonadherence, a large proportion of chronic patients could reasonably benefit from this service in a pharmacy. Second, the existence of local interprofessional networks has been identified as one of the major factors for successfully implementing a medication adherence program in community pharmacies [41, 42]. We observed at the PMU that the inclusion in the program by the physician plays a key factor. In fact, it is determinant that this program is part of the medical care plan, as many patients rely on this traditional way of therapeutic decision-making process. The other barriers that may affect program success are poor patient-pharmacist communication (resulting in an insufficient promotion of the program), difficulty in integrating the program into the pharmacy organisation, and insufficient pharmacist motivation [42].

In our model, only one full-time equivalent (FTE) pharmacist and one FTE technician followed up with the entire cohort of patients. However, the management of 16 patients only represents a 7% FTE for pharmacists and a 4% FTE for technicians (9 and 5% respectively for 27 patients). In the context of a pharmacy, this implies a solid management and planning of staff resources for each basic and advanced activity. Strategically, we recommend training at least two pharmacists and technicians to ensure a turnover in case of absence. Assuming an amortisation over 5 years, the doubling of trained professionals had a minimal impact on the break-even point (see Table 2).

Regarding the generalisability of this cost analysis to other contexts, the BEA depends on the national reimbursement system model. In our context, the “price” of the service corresponds to a fee-for-service plus the sale of pill boxes. The Swiss pharmacist can charge them only if the patient is simultaneously prescribed at least three chronic medications (resulting in three EMs). With a fourth chronic medication or more, the pharmacist can charge an additional polymedication check fee (48.60 CHF every 6 months). Although the number of tablets can affect adherence [40], these restrictions are debatable as chronic patients with only one drug (e.g., HIV, multiple sclerosis or cancer patients) also need the program. According to the PMU data, patients had an average of 2 EMs over their entire follow-up period [IQR: 1–3] [12]. Moreover, it is impossible to estimate the potential hidden incomes generated by the delivery of the program from the building of patient loyalty or the increasing of the medication adherence, both of which could add to the revenue.

The estimates or our analysis is not generalizable per se as it is context-dependent. However, the BEA equation (see Fig. 2) can be applied to other healthcare contexts to either calculate the price of the service or the number of patients to include in order to reach the local break-even point. For instance, Noain et al. 2017 estimated a potential medication review with follow-up service price ranged from €237 to €628 per patient a year in Spain, according to different scenarios including professional level of the service provider (pharmacist in charge/pharmacy owner), potential number of patients receiving the service (60/120/180) and mark-up applied (10/20/30/40%) [39].

Some methodological considerations should be noted when interpreting these findings. BEA is a simplistic representation of reality. Although the estimated parameters are based on the experience of the PMU and expert opinions, BEA assumed a similar variable cost for all patients. However, the pharmacy faces a variety of patients, leading to a range of variable costs in the type and extent of nonadherence issues, frequency of visits, number of EMs, duration of interview and duration of follow-up. For instance, at the PMU, patient retention in the program is longer in patients with more serious nonadherence issues than in patients with less serious issues. This repartition is nevertheless unknown in the community and different in each pharmacy setting. Prospective studies should assess this variability in real life. The major unpredicted event that occurs in the IMAP program is when patients do not show up and the visit is rescheduled. We have not taken this uncertainty into account in our model as extra-cost because the pharmacist is directed to another clinical activity when this situation happens.

## Conclusions

This pragmatic analysis can be used as a template to estimate the implementation costs and assess the economic feasibility and viability of a professional pharmacy service from the provider’s perspective. In a Swiss context, the implementation in community pharmacies of the described interprofessional medication adherence program for chronic patients treated with at least three medications seems to be profitable; the current medication adherence support fee-for-service system allows pharmacists to reach a break-even point with at least 13 patients. These findings are important to community pharmacists in deciding whether to implement the service and can guide their business plans and implementation strategies. The replication of similar analyses in other settings and countries is paramount for a better understanding of the feasibility and implementation costs of medication adherence programs, as well as other advanced services, in routine pharmacy care. Implementation research can also aims to optimize the service design and process to reduce its cost.

## Abbreviations

BEA: Break-even analysis
CHF: Swiss francs
EMs: Electronic monitors
FTE: Full-time equivalent
PMU: Policlinique médicale universitaire
